# AMiCUS 2.0—System Presentation and Demonstration of Adaptability to Personal Needs by the Example of an Individual with Progressed Multiple Sclerosis

**DOI:** 10.3390/s20041194

**Published:** 2020-02-21

**Authors:** Nina Rudigkeit, Marion Gebhard

**Affiliations:** Group of Sensors and Actuators, Department of Electrical Engineering and Applied Physics, Westphalian University of Applied Sciences, 45877 Gelsenkirchen, Germany; marion.gebhard@w-hs.de

**Keywords:** assistive technology, multiple sclerosis, tetraplegia, motion sensors, human-machine interaction, robot control, head control, inertial sensors, assistive robotic manipulator

## Abstract

AMiCUS is a human–robot interface that enables tetraplegics to control an assistive robotic arm in real-time using only head motion, allowing them to perform simple manipulation tasks independently. The interface may be used as a standalone system or to provide direct control as part of a semi-autonomous system. Within this work, we present our new gesture-free prototype AMiCUS 2.0, which has been designed with special attention to accessibility and ergonomics. As such, AMiCUS 2.0 addresses the needs of tetraplegics with additional impairments that may come along with multiple sclerosis. In an experimental setup, both AMiCUS 1.0 and 2.0 are compared with each other, showing higher accessibility and usability for AMiCUS 2.0. Moreover, in an activity of daily living, a proof-of-concept is provided that an individual with progressed multiple sclerosis is able to operate the robotic arm in a temporal and functional scope, as would be necessary to perform direct control tasks for use in a commercial semi-autonomous system. The results indicate that AMiCUS 2.0 makes an important step towards closing the gaps of assistive technology, being accessible to those who rely on such technology the most.

## 1. Introduction

### 1.1. Multiple Sclerosis

Multiple sclerosis (MS) is a chronic, progressive disease, in which the immune system attacks the protective sheath that covers the nerve fibers in the central nervous system (CNS), causing communication problems between the brain and the rest of the body. The resulting symptoms may differ widely, depending on the amount of nerve damage and on which nerves are affected.

MS is the most common non-traumatic cause of disability in young adults [1]. In 2013, approximately 2.3 million people were affected by the disease worldwide, resulting in a prevalence of 0.033%. While MS is present in all regions of the world, a great variation of prevalence can be observed. That means, developed countries are generally more affected [2]. Around two-thirds of those diagnosed with MS are female. In most cases, multiple sclerosis is diagnosed between the ages of 20 and 50 [3]. The common first symptoms include fatigue, impaired coordination, balance problems, numbness and/or vision disorders. However, the range and severity of possible symptoms can vary broadly. An overview of frequent symptoms can be found in [4]. Similarly, the progression of MS is highly individual and therefore unpredictable: Symptoms may occur in clearly defined phases and subside completely or partially, or gradually exacerbate over time. Despite this unpredictable nature, four general disease courses have been identified in MS as described in [5].

The majority of people with MS do not become severely disabled: Two-thirds of the people living with MS remain able to walk, though possibly requiring walking aids. However, in rare cases, MS may lead to paralysis of all four limbs, a condition also denoted as tetraplegia. Being unable to move the limbs, tetraplegics heavily depend on caretakers and assistive technology. Therefore, interfaces enabling them to control an assistive robotic arm are of major importance to at least partly restore their autonomy, and substantially improve their quality of life.

One of the biggest challenges when developing assistive systems for tetraplegic MS patients is the fact that this user group is not only severely small but also extremely heterogeneous, making any development solely for this user group economically unfeasible. Moreover, many tetraplegic MS patients have very limited financial resources as their condition commonly leads to a withdrawal from working life. For this reason, tetraplegic MS patients have no choice other than using assistive devices that have been designed for individuals with high levels of spinal cord injury, who typically do not exhibit impairments other than the paralysis itself.

### 1.2. Assistive Systems for Tetraplegics

As the control capabilities of tetraplegics are severely limited, the most natural choice is to design related assistive systems in a way that the control of the assistive robotic arm is automated as much as possible. Practically, this means that the user generates discrete control commands that lead to the execution of high-level tasks, such as grasping a book or opening a fridge. This method of control is very easy for the user. However, the experience with the assistive robot FRIEND [6] has shown that purely autonomous control still lacks the required robustness that is necessary for a commercially available assistive system, in particular in complex and changing environments. Higher robustness may be achieved by acquiring data for a large number of different situations, increasing the algorithm complexity, and/or using additional or better sensors. Until now, this had made the realization of sufficiently robust autonomous systems uneconomic.

Additionally, Kim et al. determined that the user acceptance for purely autonomous assistive systems was lower than for semi-autonomous systems [7]. This is because purely autonomous systems strictly define which actions are possible and how they are executed, limiting the freedom of the user to explore and experiment. For this reason, we come to the conclusion that direct control interfaces for tetraplegics are needed in order to restore true autonomy. Such interfaces may be used as standalone systems or to enable direct control in a semi-autonomous system. In the latter case, autonomous control may be used to perform recurring tasks and to cover long distances, while direct control may be used to intervene in critical situations, to perform correction steps, and to execute new tasks.

### 1.3. Direct Control Interfaces for Tetraplegic MS Patients

Interfaces, which use the remaining capabilities of tetraplegics to generate signals suitable for proportional real-time control, may be based on neural activity [8,9], electromyography (EMG) [10,11], gaze [12], tongue movement [13], or head motion [14,15]. While all these solutions are generally feasible to enable robotic arm control, they might not be accessible to a subset of tetraplegic MS patients, due to the presence of interfering symptoms. For example, invasive neural interfaces, which allow intuitive robot arm control, typically use brain signals recorded from the motor cortex to generate control signals. This requires intact neural function, which might not be the case for individuals with MS. In order to use EMG-based interfaces, the user must be able to contract certain muscles or muscle groups voluntarily. This involves higher levels of mental and physical effort, and is therefore unsuitable for a great percentage of MS patients who suffer from fatigue and/or weakness. Gaze-based interfaces also require considerable concentration when used for direct robot control. Though the tongue-based interface presented by Struijk et al. seems generally promising, it might not be accessible for individuals with MS who have disorders related to tongue movement.

It is important that a solution is also available for these individuals to ensure that no one falls through the cracks of assistive technology. Our previous experience with the head motion-based assistive system AMiCUS [16] makes us confident that this system can be adapted in a way that fits a wide range of tetraplegic MS patients and possibly other tetraplegics with similar symptoms. By taking the common limitations of individuals with MS into account when developing the next version of AMiCUS, we aim to bypass the problem that true personalization is not economically feasible. Table 1 summarizes some common symptoms of MS and the resulting requirements for a head motion-based assistive system, such as AMiCUS.

In the following, we present the AMiCUS system and describe in detail which changes have been made to address the limitations of version 1.0 and to incorporate the additional requirements coming from tetraplegic MS patients. Then, we describe the experimental setup that has been used to demonstrate the accessibility of AMiCUS by the example of an individual with progressed multiple sclerosis. This work closes with the conclusions and outlook for future research.

## 2. Assistive System AMiCUS

In our research group, we have developed the AMiCUS system, which is the first motion sensor-based interface that uses solely head motion to produce all the necessary signals and commands for real-time control of an application with more than three degrees of freedom (DOF), such as a robotic arm. In our previous work, we investigated two different versions of the system: a gesture-based interface, as presented by Jackowski et al. [17], and a cursor-based interface, as presented in [16]. Based on our results, we have concluded that the gesture-based interface was promising as a hands-free interface for people without head motion limitations, for example in a human–robot collaborative workplace if the hands of the human co-worker are not available for robot control.

In contrast, the cursor-based interface has been considered more suitable for individuals with head motion restrictions due to the higher accessibility and capability to adapt to special needs. However, the control structure of the cursor-based system made use of a head gesture, which was difficult to perform for many subjects and resulted in increasing neck discomfort over time. That means, the head gesture was a limiting factor for both accessibility and long-term operability. For this reason, we decided to remove the head gesture, which made a complete redesign of the control structure necessary. In the following, we briefly present the first version of the cursor-based system, here denoted as AMiCUS 1.0, and summarize the results of its evaluation in a user study [16], which served as input for the next development cycle. Then, the changes that resulted in AMiCUS 2.0 are described in detail.

### 2.1. AMiCUS 1.0

AMiCUS is a head motion-based assistive system that enables intuitive real-time control of a robotic arm. Visual feedback—for example, about the user’s head orientation or relevant system states—is given on a graphical user interface (GUI). Head motion is measured by a motion sensor system, which is placed on the head of the user. Neck flexion/extension (pitch), neck bending (roll), and neck rotation (yaw) provide three independent control signals. These signals are used to control the pose of the robot arm, i.e., the orientation and position, and to open or close the attached robotic gripper.

**Figure 1:**
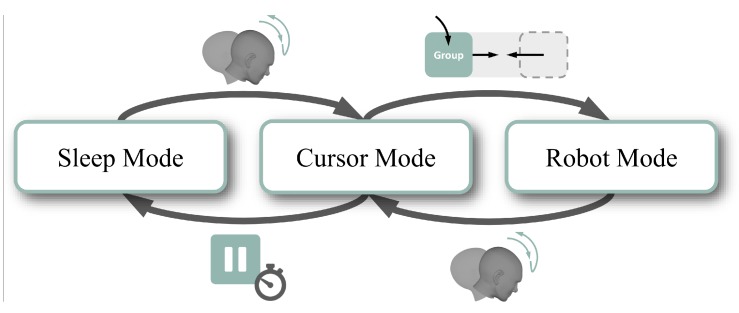
Control structure of Adaptive Head Motion Control for User-Friendly Support (AMiCUS) 1.0.

As the robot has more DOFs than available control signals, the robot DOFs are arranged in groups, each group containing no more than three DOFs. In this way, the user can control a maximum of three DOFs of the robot simultaneously. The default mapping of AMiCUS contains four different groups, namely Orientation, Vertical Plane, Horizontal Plane, and Gripper. To control the whole robot, switching between these groups is necessary. This is realized using a two-staged approach (Figure 1): within a robot group (Robot Mode), the user has to perform a switching command to leave the current group and enter Cursor Mode. In AMiCUS 1.0, this switching command is a nodding-like movement, in the following it is denoted as Head Gesture.

Within Cursor Mode, the user interacts with the GUI of AMiCUS by moving a mouse cursor using head motions. One button for each robot group is displayed on the GUI. A robot group is entered by activating its corresponding button. In AMiCUS 1.0, these buttons are implemented as Slide Buttons. Dwell Buttons are used to access menu items, for example to calibrate the system or to enter Sleep Mode. In Sleep Mode, both the cursor and robot control are deactivated, allowing the user to rest. The system returns to Cursor Mode after a correct execution of the switching command.

In [16], AMiCUS 1.0 is described in detail and the results of a user study with healthy and tetraplegic subjects are presented. Overall, the results of the user study were promising: All users could operate the robot, even those with severe head motion limitations. Moreover, user acceptance—which is often a critical factor—was very high. However, we have noticed that the Head Gesture was demanding, in particular for the tetraplegics, and caused neck discomfort over time. For this reason, we decided to remove the Head Gesture from the system. Table 2 summarizes all possibilities for improvement, which have been identified in the user sudy, in the form of requirements for the next prototype, AMiCUS 2.0.

It has never been explicitly evaluated how well AMiCUS 1.0 met the needs of tetraplegic MS patients according to Table 1. However, Appendix A gives an overview of some system features of AMiCUS 1.0 and how they relate to the needs of this user group. From the results of the user study, we can infer that ease of control (Requirement 1.1) may be improved further by facilitating gripper rotations, increasing the intuitiveness of the depth control, and possibly simplifying the switching process. Furthermore, Requirement 1.6 may be addressed by redesigning the switching process to include only slow and smooth head motion, increasing the control ergonomics. Requirements 1.2, 1.3, and 1.5 have not been addressed in the user study carried out with AMiCUS 1.0. Requirements 1.4 and 1.7 are immanently met in the system.

### 2.2. AMiCUS 2.0

The main research challenge when designing AMiCUS 2.0 was developing an alternative control structure without head gestures in a way that usability was at least as high as in the previous version. On top of that, we aimed at meeting the requirements listed in Table 1 and Table 2 as effectively as possible. In order to address all requirements, a multitude of changes have been made, many of these being interdependent. We have decided to cluster the changes into five different groups, addressing the following criteria:

1. Usability of the system calibration.
2. Ergonomics, ease, and efficiency of group switching.
3. Ergonomics and usability of the GUI.
4. Intuitiveness of robot control.
5. Adaptibility to the skill level of the user.

In the following, the changes in the new version and the underlying motivations are presented with respect to these criteria.

#### 2.2.1. Usability of the System Calibration

In AMiCUS 1.0, an assistant has to start the initial Cursor Calibration, requiring coordination with the user. After successful calibration of the cursor, the user has to manually initiate the Robot Calibration. Possible re-calibrations can be started by the user but the Cursor and Robot Calibration have to be started separately and the procedures have to be gone through completely. This has low user-friendliness given the two main uses:

1. Startup: After startup, all calibration procedures have to be fully completed.
2. Drift: In case of sensor drift, only the zero position needs to be re-calibrated.

In AMiCUS 2.0, the calibration processes have been modified with respect to these cases in order to increase the calibration usability. The first case has been addressed by starting the Robot Calibration automatically after completion of the Cursor Calibration. Furthermore, the user can start the initial Cursor Calibration independently by tilting the head up. To address the second case, a separate Offset Calibration has been introduced, allowing the user to re-calibrate solely the zero position.

#### 2.2.2. Ergonomics, Ease, and Efficiency of Group Switching

Based on the results of our user study, the Head Gesture achieved low user acceptance. One reason was that the quick head movements required for correct gesture execution were physically demanding [18], causing neck discomfort during long-term use. Moreover, the Head Gesture imposed higher requirements on the head motion capabilities of the user than the rest of the system [17], making it a limiting factor for the accessibility of AMiCUS. Consequently, we decided to replace the Head Gesture to increase switching ergonomics and system accessibility.

**Figure 2:**
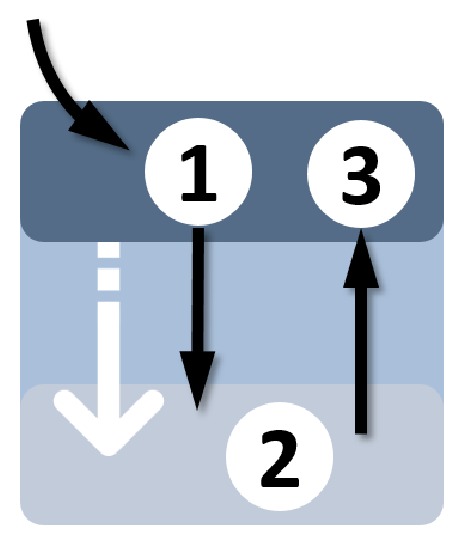
Working principle of the Vertical Slider: For successful activation, one has to dwell inside the slider until visual feedback is given (1). Then, one has to move the slider down to the bottom of the deadzone (2) and back to the initial position (3).

The resulting main requirement for the new switching element was that it required solely smooth and slow head motion, which was clearly distinguishable from head motion used for direct robot control. This was very challenging because previously onset acceleration had been used to separate a switching command (Head Gesture) from direct control signals. Several different ways of switching have been implemented and tested; however, eventually we decided to increase the size of the deadzone and place a slide button inside, as shown in Figure 2. One reason for this choice was that our previous research had already shown high usability and accessibility for this kind of control element. However, compared to the Slide Button in Cursor Mode, the button here was rotated by 90°. Therefore, we denote it as Vertical Slider. Please note that increasing the deadzone made voluntary head motion inside the deadzone possible. At the same time, the head range of motion (ROM) effectively available for the robot control was reduced. For this reason, it is important to make the deadzone only as big as needed to enable Vertical Slider activation.

**Figure 3:**
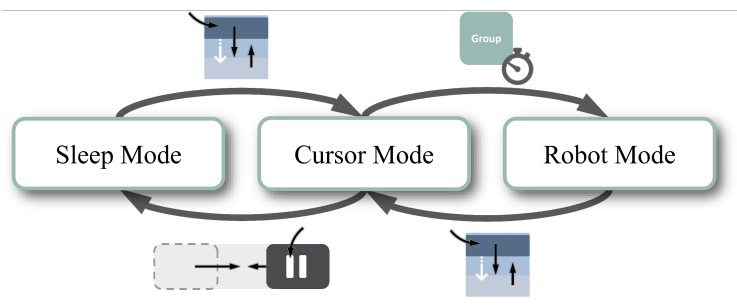
Control structure of AMiCUS 2.0. Compared to AMiCUS 1.0, the Slide Buttons and Dwell Buttons have been swapped in Cursor Mode. The Head Gesture has been replaced by the Vertical Slider.

In the Cursor Mode, the Slide Buttons were replaced by Dwell Buttons. This was done to simplify the overall switching process and make it as fast as possible while maintaining robustness. The resulting control structure of AMiCUS 2.0 is shown in Figure 3. Please note that the changes to the control structure made a redesign of the GUI necessary.

#### 2.2.3. Ergonomics and Usability of the GUI

**Figure 4:**
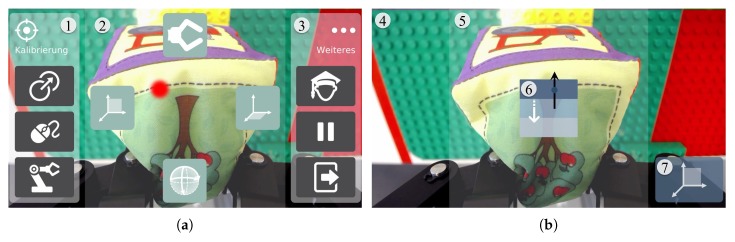
The GUI of AMiCUS 2.0 in Cursor Mode (**a**) and Robot Mode (**b**), respectively.

Figure 4 shows the GUI of AMiCUS 2.0 during Cursor Mode (a) and Robot Mode (b). In both modes, the interface has been designed axisymmetrically to increase the ergonomics during control.

In Cursor Mode, the traditional mouse cursor has been replaced with a big red dot to increase its visibility, particularly benefiting users with impaired eyesight. The Dwell Buttons for each of the four robot groups are positioned at equal distances around the screen center (2). The symmetrical head movements with low head deflection angles, which are required for group selection, improve ergonomics. The position of the buttons always remains the same, so that a certain head movement is directly associated with entering a certain group. This way, the mental workload is minimized.

In the left pane, the user can access the calibration menu (1). The calibration menu contains Slide Buttons for Offset Calibration, Cursor Calibration, and Robot Calibration (from top to bottom). The Cursor and Robot Calibration have been presented in [16]. The Offset Calibration is new in AMiCUS 2.0, as described in Section 2.2.1. Another menu (3) is placed in the right pane and contains Slide Buttons to switch between the newly introduced Beginner’s and Advanced Mode, to enter Sleep Mode and to exit the program (from top to bottom). During Sleep Mode, neither the cursor nor the robot arm can be controlled. That means, the program is paused, allowing the user to take a break and recover, if necessary. The user can resume control independently by activating a Vertical Slider (Figure 3).

In both Cursor and Robot Mode, the image of the gripper camera is shown in the background, filling the whole screen (4). Feedback about the current head orientation of the user is shown in area (5), containing the deadzone with the Vertical Slider (6). An icon of the current robot group is displayed in the bottom right corner of the screen (7), illustrating the control options of the current group. Note that AMiCUS 2.0 no longer makes use of submenus, therefore showing all menu items at a glance and allowing direct access of each item. This has been done to reduce the mental load and facilitate menu item access.

#### 2.2.4. Usability of Robot Control

According to the feedback obtained during the user study in [16], the majority of users found the depth control of the robot arm not intuitive using AMiCUS 1.0. That is, they wanted the robot to move towards them when they tilted the head down, and away from them when they tilted the head up. For this reason, the mapping of the pitch DOF of the head (θ) onto the robot DOF for depth control (*z*) has been inverted in AMiCUS 2.0.

**Figure 5:**
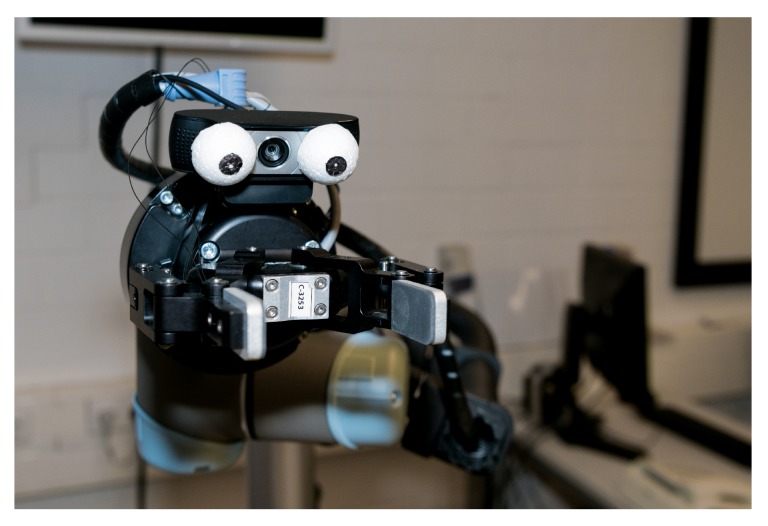
A robotic gripper with dummy eyes to faciliate mentally mapping head motion onto gripper rotations.

In AMiCUS 2.0, the appearance of the robotic gripper has been modified to resemble a face by attaching dummy eyes to the gripper camera as shown in Figure 5. This has been done to facilitate mentally mapping the head motion onto gripper rotations, therefore increasing the control intuitiveness. The detailed motivation for this specific modification is given in Appendix B.

#### 2.2.5. Adaptability to the Skill Level of the User

To satisfy a wide range of users, AMiCUS has to be able to accommodate the needs of users with various skill levels. In our user study [16], for example, we have observed that some users were skilled at performing 3D rotations and found the direct robot control too slow, while others found the control speed satisfactory but struggled performing the 3D gripper rotations. Since these opposing requirements can hardly be accommodated within a single system implementation, AMiCUS 2.0 offers dedicated control modes for two different user groups:

1. Beginner’s Mode: Designed to minimize both mental and physical demands for users with restricted capabilities to control the robot arm and/or for untrained users.
2. Advanced Mode: Optimized for high control efficiency, aiming at users with high spatial imagination and no or mild head movement limitations.

We have observed that first-time users often could not benefit from the possibility to perform simultaneous rotations around multiple axes; the main reason being their limited capability to imagine such complex rotations. As a consequence, most users tried to simplify the task by rotating the gripper around a single axis at a time. That means, all rotations around multiple axes were undesired. Therefore, it was the most natural choice to disable simultaneous rotations completely in Beginner’s Mode. In Advanced Mode, however, simultaneous rotations are still possible, offering high control efficiency. For the same reason, the maximum velocity of all robot DOFs has been increased.

### 2.3. Mappings for Users with Less than Three DOFs of the Head

The default mapping between head and robot motion of AMiCUS assumes that the head ROM is sufficient to control three robot DOFs proportionally and simultaneously. However, this is not always the case: For individuals with very high levels of spinal cord injury (SCI) or as result of a progressive disease, such as MS, the head ROM may be impaired to such an extent that one or more DOFs are not or only partially available for control. If a DOF is generally available, but not usable for proportional control, it may still be used for discrete robot control, that is, at a single speed.

However, in the event of missing head DOFs, the mapping needs to be modified: In the case where only one head DOF is available, this DOF is used to control all robot DOFs sequentially. Note that, in this case, cursor control also requires adaptation, as discussed in Appendix C. If two head DOFs are available, each robot group must contain a maximum of two robot DOFs. Thus, the Orientation group of the default mapping is split into two groups. Arbitrary mappings between the head and robot DOFs may be realized; however, for the sake of intuitiveness, we propose the following: A missing roll DOF (ϑ) is substituted by the yaw DOF (ψ), and vice versa. In case the pitch DOF (φ) is not available, it is substituted by the head DOF that is unused in the respective group of the default mapping.

**Figure 6:**
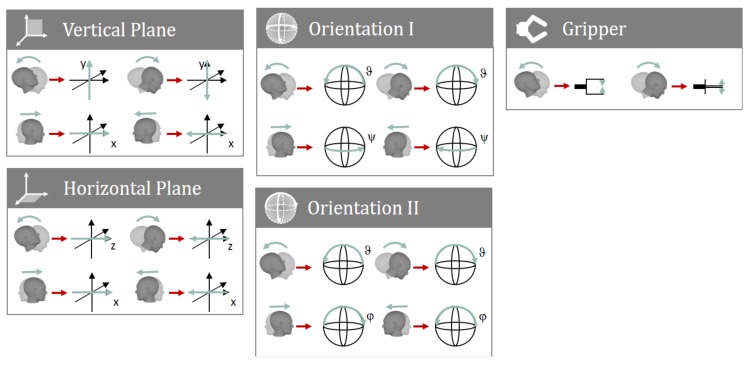
The proposed mapping of AMiCUS 2.0 in the case where the roll DOF cannot be used for control.

Figure 6 shows the proposed mapping if the roll DOF is unavailable. Here, the Orientation group is split into two groups, i.e., Orientation I and Orientation II, with two DOFs each. In both groups, the pitch DOF (ϑ) of the head controls the pitch DOF of the robot, whereas the yaw DOF (ψ) of the head controls the robot’s yaw DOF in the Orientation I group and the robot’s roll DOF in the Orientation II group.

To conclude, the mapping can be adapted easily to accommodate a wide range of head motion restrictions. On one hand, this increases the number of possible users, likely making the system more profitable. On the other hand, it allows AMiCUS to accompany users with progressive diseases for a long time and during many possible phases. In this way, users do not need to change assistive systems when their control abilities degrade temporarily or permanently. If desired, AMiCUS may also be programmed to automatically adapt to varying head ROMs in order to avoid emphasis on progressive impairments.

## 3. Materials and Methods

### 3.1. Research Goals

Within this work, we have introduced AMiCUS 2.0. The goal of AMiCUS 2.0. is to make a gesture-less interface for intuitive real-time robotic arm control available, which has at least as high usability as AMiCUS 1.0. Additionally, we want to provide a proof-of-concept that AMiCUS 2.0 is accessible to tetraplegics with additional impairments.

### 3.2. Test Subject

For our experiments, we chose to include the test subject who had the most severe head motion limitations of all tetraplegics that have participated in our experiments so far. Among those, this subject was additionally the only one who suffered from further symptoms that were relevant with respect to the accessibility of an assistive system. Therefore, if the abilities of this subject are sufficient to control the robotic arm, we can directly infer that all subjects from our previous experiments will be able to control the robot as well.

The chosen test subject was female and 58 years old. At the time of the experiments, all four limbs were paralyzed. Moreover, lateral flexion of the head was restricted to an extent that the ROM was within the range of unintended motion. For this reason, it has been decided that this DOF was unsuitable for robot control. The mapping shown in Figure 6 was used for control.

Further symptoms, which were relevant for operating AMiCUS, were fatigue/attention deficit and visual impairment. The test subject chose not to correct the latter using visual aids. A Landolt eye test was carried out prior to the experiments and yielded 0.4 to 0.5 visual acuity. This corresponds to 40% to 50% of the visual acuity that is defined as unimpaired. On top of that, the test subject could not identify numbers on color plates during an Ishihara color vision test, indicating that color vision may be impaired as well. However, we want to emphasize that these results are purely indicative and that a proper eye test can only be carried out by a specially-trained ophthalmologist.

### 3.3. Experiments

For this work, two experiments have been set up: The first experiment aimed at finding out whether AMiCUS 2.0 was able to provide intuitive real-time control of a robotic arm, while being at least as user-friendly as AMiCUS 1.0. To this end, the subject tested both versions and compared them in terms of usability. As AMiCUS 1.0 was not accessible to our subject in its default form, the system has been customized to meet her needs. More specifically, the parameters for the Head Gesture have been adapted and the Orientation group has been split as described in Section 2.3.

In the second experiment, the test subject carried out an activity of daily living (ADL). using AMiCUS 2.0 in a semi-realistic scenario. We wanted to show that a tetraplegic MS patient was able to operate the robotic arm in a temporal and functional scope, as would be necessary for use in a semi-autonomous system.

The subject used AMiCUS 2.0 in Beginner’s Mode in both experiments due to her MS-related impairments.

#### 3.3.1. Comparison of AMiCUS 1.0 and 2.0

In this experiment, AMiCUS 1.0 and 2.0 have been compared with respect to the criteria presented in Section 2.2 (Note that—with a single user—the adaptibility to various user skill levels could not be tested).

The experimental setup was as follows: The test subject was seated in front of an empty table with a direct view of the robot arm and screen of AMiCUS. The spatial setup in terms of the screen, robot, table, and user positioning was identical to the setup described in [16].

As part of the experiment, both AMiCUS 1.0 and AMiCUS 2.0 were tested for 20 min each. The test user was instructed to enter each robot group twice. After entering a group for the first time, she was allowed to freely move the robot according to its control possibilities within the called group. After the second call, she was instructed how to move the robot. That is, she was told in which direction she was supposed to move the robot arm and when to stop. All group switches were announced by the experimenters. After every group had been called twice, the test user was asked to enter Sleep Mode and Robot Calibration, two times each. This experimental protocol allowed the test user to assess all system functionalities that were subject to change between the two tested AMiCUS versions.

The test user has been encouraged to voice her thoughts during the task, applying the Think Aloud Method [19]. After the experiment, the test subject completed an evaluation sheet for comparison of both AMiCUS versions. The evaluation sheet contained 21 statements that could be rated with whole numbers between 1 (“I strongly disagree.”) and 5 (“I strongly agree.”) as shown later with the results in Table 4. The statements were chosen to obtain information regarding the impact of each change being made. It is worth noting that the user feedback had a mainly validating purpose. This is because the implemented changes were either trivial, thus being obvious improvements, or because other supporting information was available. The first case was, for example, true for the changes made to the calibration. An example for the latter case is the Vertical Slider, which is a rotated version of the Slide Button that has been extensively tested in a previous user study [16].

To obtain more detailed feedback about the main changes, the test subject was additionally asked four open questions, as presented in Table 3. The entirety of the user feedback was grouped according to the criteria listed in Section 2.2. As adaptibility could not be tested, changes to the system resulting from the introduction of Beginner’s Mode have been assigned to the category “usability of robot control”.

#### 3.3.2. Evaluation under Semi-Realistic Conditions

In this experiment, the subject carried out an ADL using AMiCUS 2.0 in a semi-realistic scenario. The spatial arrangement was identical to the previous experiment. A miniature refrigerator with inner dimensions of 20 cm by 30 cm was placed on the left side of the table. The fridge was completely open, the hinge side pointing away from the user. An opened 0.5 L bottle filled with water was placed inside of the refrigerator. A conventional water glass was positioned on the table in front of the test subject. The task of the user was to grasp the bottle, pour water into the glass, and then place the bottle next to the glass on the table. Again, the subject was instructed to voice her thoughts, following the Think Aloud Method.

Note that simplifying the task by choosing the bottle and refrigerator to be open did not limit the system’s applicability in real life as bottle pourers and remote controlled door openers are commercially available to assist a person with motion-impairment, if needed.

## 4. Results and Discussion

### 4.1. Comparison of AMiCUS 1.0 and 2.0

In the following, the results of the experiment with respect to aforementioned criteria are presented.

#### 4.1.1. Usability of the System Calibration

As shown in Table 4, the test user fully agreed that all changes made to the calibration procedure were improvements compared to the old version (Statements 1–4). It is worth noting that the default used ROM to control the robot was feasible for the test user, even though she suffered from severe motion restrictions of the (unused) roll DOF.

#### 4.1.2. Ergonomics, Ease and Efficiency of Group Switching

With respect to the switching process (Statements 5–10), the test user found the Vertical Slider much better than the Head Gesture. She remarked that she did not find the Head Gesture stressful but thought that it was uncomfortable during long-term use. For this reason, she preferred the gesture-less switching procedure. Furthermore, she liked the Dwell Button much better than the Slide Button and found the overall switching process in AMiCUS 2.0 easier but equally fast as in AMiCUS 1.0.

For the switching times, we make the following theoretical considerations: The switching process of AMiCUS 1.0 contains the Head Gesture and the Slide Button, while AMiCUS 2.0 contains the Dwell Button and the Vertical Slider. That means, both versions make use of a slide button. In general, vertical head movements (Vertical Slider) are easier and quicker to perform than horizontal ones (Slide Button) [20], so that the Vertical Slider should outperform the Slide Button. In this case, however, the Vertical Slider was displayed smaller on the GUI than the Slide Button, negatively affecting its ease of use [21]. In practice, this may result either in slightly lower success rates or activation times of the Vertical Slider.

The second switching element of AMiCUS 1.0 is the Head Gesture. The execution time of the Head Gesture is enforced to range from 0.5 s to 1.5 s. In contrast, the second switching element of AMiCUS 2.0, the Dwell Button, has been tested with a dwell time of 2 s. As a result, we expect the switching process of AMiCUS 2.0 to be marginally slower than the one of AMiCUS 1.0. This is in line with the perception of our test user. The longer activation time, however, has been traded with increased ergonomics and higher accessibility. This explains why the subject still preferred the switching process of AMiCUS 2.0. Furthermore, taking learning effects and the expected performance increase after parameter tuning of the Vertical Slider into account, we assume the success rate of the switching process will be higher for AMiCUS 2.0.

The test user also fully agreed that possible differences with respect to success rates and switching times between AMiCUS 1.0 and 2.0 might disappear due to learning effects. She replied to the open questions 1 and 2 (Table 3) that she liked the simplicity and playfulness about the new switching process. She appreciated that the visual and acoustic feedback stimulated multiple senses. Overall, she positively described the new switching process as “Robot control for beginners”. She did not have any points of criticism.

Overall, the results are in line with the results of our previous user study [16], in which the Slide Button outperformed the Head Gesture, even though its activation time was longer.

#### 4.1.3. Ergonomics and Usability of the GUI

Comparing the GUIs of AMiCUS 1.0 and 2.0 (Statements 11–17), the test user found both GUIs equally good overall. She remarked that she had already liked the GUI of AMiCUS 1.0 very much, so that there was little room for AMiCUS 2.0 to be better. The subject agreed that keeping overview of the menu was much better in the new version, but accessing items was not necessarily easier.

The latter is supported by theoretical considerations: In AMiCUS 1.0, some menu items are accessed directly via Dwell Buttons, while others are nested, requiring a cascade of Dwell Button activations. In contrast, for AMiCUS 2.0, all menu items are accessed directly via Slide Buttons. Thus, menu item access is only easier for previously nested menu items but not for the menu items that were already directly accessible with AMiCUS 1.0.

Replying to open questions 3 and 4, she used again the phrase “Robot control for beginners” to describe what she liked about the new menu. She did not have any points of criticism or suggestions for improvement. The test user neither found cursor control easier with the new cursor, nor could she generally see the cursor better. However, she remarked that the cursor was a lot better to find on the screen. When asked if she liked the bigger image of the gripper camera better, she answered with an anecdote, saying that she did not like the bigger image better because it showed such level of detail that she was “shocked” when the camera was facing her.

#### 4.1.4. Usability of Robot Control

As mentioned earlier, the size of the deadzone has been increased in AMiCUS 2.0 to allow placing the Vertical Slider inside. As a result, the available range of motion used for robot control became smaller, slightly increasing the control sensitivity. At the same time, the control precision decreased. The test user, however, did not experience any negative effects due to the increased deadzone size.

With respect to the ease of control, she agreed that the depth control was much more intuitive in AMiCUS 2.0. Moreover, she agreed that attaching dummy eyes to the gripper helped imagining rotations. She could not tell whether performing rotations was easier when simultaneous rotations were disabled. This may be due to the fact that she was used to making sequential rotations using both versions.

### 4.2. Evaluation under Semi-Realistic Conditions

**Figure 7:**
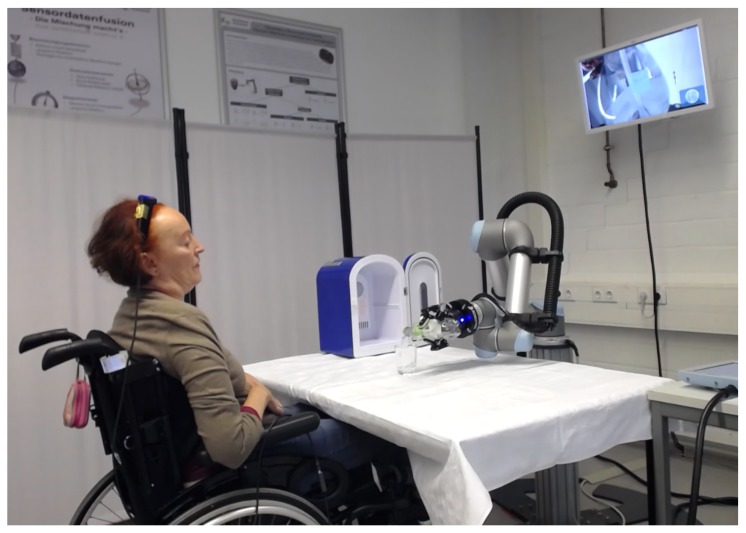
The test subject uses AMiCUS 2.0 to pour water from a bottle into a glass.

The user could solve the task independently, that is, without requiring help from another person. Figure 7 shows how the test subject pours water into the glass, providing the proof-of-concept that AMiCUS generally has the potential to be used for tasks with high complexity as required for a commercially available assistive system used in a home environment.

However, minor improvements are still possible: The experimenters observed that the subject had difficulties making sense of the camera image when the gripper was not pointing upright. Furthermore, the test user tended to perform incorrect head movements when looking at the camera image while trying to control the robot within a group using world coordinates. Therefore, future versions are likely to benefit from automatic rotation of the camera image, as is similarly done for endoscopic images [22]. In contrast to the proposal of Lee et al., data from the force/torque sensors of the robot arm may be used instead of accelerometer and gyroscope data.

Another observation was that the subject sometimes did not notice when the robot control was deactivated after a quick movement, resulting in confusion about the robot arm not responding to her head motion. Consequently, it should be indicated more clearly when the robot control is deactivated. This could be achieved by graying out the GUI, as is commonly done in computer programs. However, all these issues are considered to be minor, and not limiting the further development of AMiCUS into a commercially available assistive system.

## 5. Conclusions and Outlook

In this section, the experimental results are summarized, followed by the conclusions with respect to system accessibility for tetraplegic MS patients and their implications for future work.

### 5.1. Summary of Results

Within the experiments, the subject confirmed that all changes to the calibration procedure increased usability. Furthermore, we have shown that AMiCUS 2.0 provides a functional gesture-free control structure with higher usability but slightly longer switching times than AMiCUS 1.0. Overall, the usability of the GUI of AMiCUS 2.0 was assessed at least as high as for AMiCUS 1.0. The subject confirmed that keeping overview of the menu was much better in the new version. Moreover, the subject found that intuitiveness of the robot control was higher for AMiCUS 2.0. Most importantly, she did not perceive any negative effect on the precision of the robot control due to the expansion of the deadzone. Based on our results, further modification of AMiCUS 2.0 is not necessary at the moment. Future research will investigate the ergonomics of AMiCUS 2.0 during long-term use, including EMG measurements on a multitude of subjects.

### 5.2. Accessibility for Tetraplegic MS Patients

As part of this work, the accessibility of AMiCUS 2.0 for a tetraplegic MS patient has been demonstrated in a temporal and functional scope, as would be necessary for the use of AMiCUS 2.0 in a semi-autonomous system. This way, the proof-of-concept has been provided that AMiCUS 2.0 is generally feasible to meet the needs of this challenging user group. Table 5 illustrates the results in the form of a gap analysis between the AMiCUS versions and the requirements, which MS patients may impose on a head-controlled assistive system.

Overall, both versions show at most minor gaps with respect to the system requirements, giving a positive prognosis for the accessibility for individuals with multiple sclerosis. For example, both AMiCUS versions do not impose any limitations on users suffering from disturbances in feeling (Requirement 1.4) and/or speech disorders (Requirement 1.7) as tactile feedback and speech/tongue input are not used.

In direct comparison, AMiCUS 2.0 outperforms AMiCUS 1.0 when addressing memory/attention deficit (Requirement 1.1), paralysis (Requirement 1.3), and weakness (Requirement 1.6). Memory and attention deficit are addressed better in AMiCUS 2.0 due to the dummy eyes on the gripper camera and inverting the mapping for depth control of the robot. Furthermore, disabling simultaneous gripper rotations and discarding nested menu items made the system operation easier. Accessibility in the presence of (progressing) paralysis is higher for the new AMiCUS version due to the provision of additional mappings for users with less than three available head DOFs. Also discarding the Head Gesture, which imposed higher requirements on the movement abilities of the user than the rest of the control, increased the accessibility. Removing the Head Gesture additionally reduced the required physical effort for robot control, benefitting users who suffer from weakness.

In AMiCUS 2.0, usage of the full screen gripper camera image and possibly the larger cursor facilitate control for individuals with visual impairments (Requirement 1.2). At the same time, however, the full screen gripper camera image leads to decreased contrast between the interaction elements and background. Color blindness may be addressed better by changing the color scheme of the GUI. Adding high-contrast borders around interaction elements might further improve accessibility for users with visual impairments. Alternatively, color filters as provided by most standard operating systems or available for download may be used.

The system’s performance in the presence of tremors (Requirement 1.5) is currently unknown due to unavailability of suitable subjects. It is suspected that the design of special filters to remove signal noise caused by the tremor would be necessary to address this issue. Raya et al. [23] successfully used this approach to suppress unintended head motion in control signals of a human–computer interface for individuals with cerebral palsy. For future research, it would be interesting to investigate if this solution can be applied to AMiCUS as well.

Nonetheless, the current results indicate that AMiCUS 2.0 is already accessible for a wide range of tetraplegics, including individuals with MS and possibly other diseases with similar symptoms. Furthermore, a proof-of-concept has been provided that AMiCUS possesses the potential to be used for complex pick-and-place tasks, which is a prerequisite for use as a commercial assistive system in a home environment. Future work will involve a usability study of AMiCUS 2.0 in application scenarios defined by assistive system providers, and with a larger number of tetraplegic users with additional special needs.

## Figures and Tables

**Table 1:** Common multiple sclerosis (MS) symptoms and the resulting requirements for a head motion-based assistive system.

No.	Symptom	Requirement
1.1	Fatigue, memory/ attention deficit	The use of the system must be easy, intuitive, and provide the possibility to take breaks, reducing the mental effort
1.2	Visual impairment	Visual feedback should be large and with high contrast
1.3	(Progressing) paralysis	The system needs to be adaptable to the available head range of motion (ROM) and resulting control capabilities of the user
1.4	Disturbances in feeling	The system should not make use of tactile feedback
1.5	Tremor	The system must guarantee that unintended motion resulting from tremor is not used for robot control
1.6	Weakness	The system should provide the possibility to take breaks and use only slow and smooth movements for control, minimizing the physical effort
1.7	Speech disorders	The system should not use speech or tongue movement as additional input modality

**Table 2:** Requirements for AMiCUS 2.0 based on the user study results.

No.	Requirement
2.1	Ergonomics of the switching process must be improved
2.2	The switching process should be simplified, possibly being faster
2.3	The level of difficulty of the gripper rotations should be adapted to the capabilities of the user
2.4	The intuitiveness of the depth control should be improved
2.5	The speed of the robot control should be adapted to the skills of the user

**Table 3:** Open questions to assess the usability of AMiCUS 2.0.

No.	Question
1	What do you think is good about the new switching procedure?
2	What do you think is not good about the new switching procedure and how can it be improved?
3	What do you think is good about the new menu?
4	What do you think is not good about the new menu and how can it be improved?

**Table 4:** Evaluation sheet comparing AMiCUS 1.0 and AMiCUS 2.0 including results.

	No.	Statement	Rating	
**Usability of the System Calibration**	1	It is much better that I can start the calibration myself in the new version.	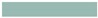	5
	2	It is much better that Robot Calibration starts automatically after Cursor Calibration in the new version.	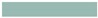	5
	3	The default size of the control area for robot control in the new version is very good.	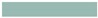	5
	4	It is very good that there is a separate Offset Calibration in the new version.	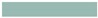	5
**Ergonomics, Ease and Efficiency of Group Switching**	5	The Vertical Slider is much better than the Head Gesture.	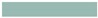	5
	6	The Dwell Button is much better than the Slide Button.	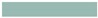	5
	7	Switching is much easier with the new version.	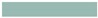	5
	8	Switching is much faster with the new version.	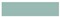	3
	9	I think it is very unlikely that a robot group will be entered unintentionally with the new version.	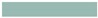	5
	10	I think that possible remaining difficulties when using the new version will be overcome due to learning effects.	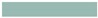	5
**Ergonomics and Usability of the GUI**	11	I like the new Cursor GUI much better than the old one.	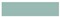	3
	12	I can keep overview of the menu much better with the new version	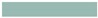	5
	13	I can access the menu points much easier with the new version.	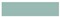	3
	14	I can see the new cursor much better than the old cursor.	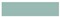	3
	15	Cursor control is much easier with the new cursor.	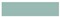	3
	16	I like the new Robot GUI much better than the old one.	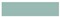	3
	17	I like the big camera image of the new version much better than the small one of the old version.	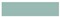	3
**Intuitiveness of Robot Control**	18	The expansion of the deadzone in the new version has no negative effect at all on the precision of the robot control.	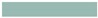	5
	19	Depth control is much more intuitive than before.	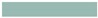	5
	20	I can imagine gripper rotations much better if the gripper looks like a face.	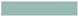	4
	21	Control is much easier without simultaneous rotations.	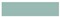	3

Rating: 1 = “I strongly disagree”, 2 = “I disagree”, 3 = “I partly agree”, 4 = “I agree”, 5 = “I strongly agree”.

**Table 5:** Gap analysis between system requirements and both AMiCUS versions.

No.	Symptom	Requirement	AMiCUS	
			1.0	2.0
1.1	Fatigue, memory/ attention deficit	The use of the system must be easy, intuitive, and provide the possibility to take breaks, reducing the mental effort	+	++
1.2	Visual impairment	Visual feedback should be large and with high contrast	+	+
1.3	(Progressing) paralysis	The system needs to be adaptable to the available head ROM and the resulting control capabilities of the user	+	++
1.4	Disturbances in feeling	The system should not make use of tactile feedback	++	++
1.5	Tremor	The system must guarantee that unintended motion resulting from tremors is not used for robot control	n.a.	n.a.
1.6	Weakness	The system should provide the possibility to take breaks and use only slow and smooth movements for control, minimizing the physical effort required	o	++
1.7	Speech disorders	The system should not use speech or tongue movement as additional input modality	++	++

**Figure A1:**
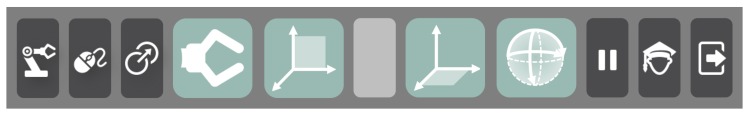
Proposal for a GUI that only requires one head DOF. Here, the example is for the yaw DOF. In the center may be a small deadzone. Robot group buttons may be close to the center, with menu items at the sides. All buttons may be implemented as Dwell Buttons. Note that this visualization is simply illustrative as the robot groups still require three head DOFs.

## Abbreviations

The following abbreviations are used in this manuscript:

ADL	Activity of daily living
CNS	Central nervous system
DOF	Degree of freedom
EMG	Electromyography
GUI	Graphical user interface
MS	Multiple sclerosis
ROM	Range of motion
SCI	Spinal cord injury

## Appendix A. How AMiCUS 1.0 Addresses the Needs of MS Patients

**1.1: Fatigue, memory/attention deficit**

*Requirement:* The use of the system must be easy, intuitive, and provide the possibility to take breaks, reducing the mental effort.

*Features:*

✓ Intuitive mapping of head DOFs onto robot DOFs
✓ Only one head gesture has to be memorized for group switching
✓ Self-explanatory GUI with optical and acoustic feedback
✓ Static positioning of robot group buttons
✓ Implementation of Sleep Mode to take breaks
× Simultaneous rotations are mentally demanding
× Nested menu items are cumbersome to access

**1.2: Visual impairment**

*Requirement:* Visual feedback should be large and with high contrast.

*Features:*

✓ Clean GUI with high contrast
✓ Large interaction elements
× Small cursor is sometimes difficult to find
× Small gripper camera image

**1.3: (Progressing) paralysis**

*Requirement:* The system needs to be adaptable to the available head ROM and the resulting control capabilities of the user.

*Features:*

✓ Calibration for available head ROM
× The Head Gesture limits system accessibility
× Mappings for users with less than three available head DOFs are not implemented

**1.4: Disturbances in feeling**

*Requirement:* The system should not make use of tactile feedback.

*Features:*

✓ Solely optical and acoustic feedback

**1.5: Tremor**

*Requirement:* The system must guarantee that unintended motion resulting from tremor is not used for robot control.

*Features:*

✓ Effect of tremor is not yet investigated

**1.6: Weakness**

*Requirement:* The system should provide the possibility to take breaks and use only slow and smooth movements for control, minimizing physical effort.

*Features:*

✓ Implementation of Sleep Mode to pause control
✓ Usage of slow and smooth control movements
× Head Gesture requires quick head movements
× Simultaneous rotations are physically demanding
× Assymmetrical GUI design creates one-sided strain of the neck

**1.7: Speech disorders**

*Requirement:* The system should not use speech or tongue movement as additional input modality.

*Features:*

✓ Control solely based on head motion input

## Appendix B. Motivation for Dummy Eyes on Gripper Camera

Imagining rotations in gripper coordinates and being able to map head motion onto gripper rotation have shown to be difficult for many untrained users. This is surprising, given the fact that humans are able to perform these mental tasks effortlessly in a different context: If a test subject sees another person in an arbitrary pose with an arbitrary head orientation, then the subject will usually be able to demonstrate immediately with her own head movements how the person has to move the head in order to end up in a certain different head orientation. It is common opinion that mirror neurons enable this capability [24], which is apparently not naturally available when mapping someone’s own motion onto the motion of something as abstract as a robotic gripper.

A similar phenomenon can be observed for the ability of identifying subtle differences: While humans are usually naturally able to perceive and interpret subtle changes in the face of a person, they will typically not be able to do the same for subtle changes between car models. However, research has shown that car experts use the same area of their brain to identify cars that is normally used for face recognition [25], showing that this brain area is not dedicated to faces only, as believed previously. This indicates that training may also allow users to intuitively map their head motion onto the rotations of a robotic gripper, as the general capability to perform this kind of action is already present in the human brain. This is in line with our experience with trained users. However, changing the outer appearance of the gripper to resemble a face may facilitate involving the brain areas, which are capable of mirroring the head rotations of another human.

## Appendix C. Cursor Control with a Single Head DOF

In the case where only one head DOF is available for control, the default GUI of AMiCUS cannot be used. It is, however, possible to redesign the GUI in a way that a single head DOF is sufficient. All interaction elements may be aligned in a row or column, depending on which head DOF is available. For example, if the yaw-DOF is available, all buttons may be arranged along a horizontal line, with the buttons for the robot groups preferably close to the center of the line (Figure A1). Slide Buttons may still be used. Note that they have to be implemented as Vertical Sliders if the available DOF is the pitch DOF. However, based on prior experience, the authors suggest that the usage of Dwell Buttons might provide higher usability for single-DOF GUIs.
